# Wild Insects Contribute More to Mango Pollination and Yield than Exotic Honeybees During Induced Off-Season Flowering in Southern Mexico

**DOI:** 10.3390/plants15071124

**Published:** 2026-04-07

**Authors:** Rodrigo Lucas-García, Víctor Rosas-Guerrero, Eduardo Cuevas, Carina Gutiérrez-Flores

**Affiliations:** 1 Posgrado en Recursos Naturales y Ecología, Facultad de Ecología Marina, Universidad Autónoma de Guerrero, Acapulco de Juárez 39390, Mexico; 2 Escuela Superior en Desarrollo Sustentable, Universidad Autónoma de Guerrero, Tecpan de Galeana 40900, Mexico; 3 Laboratorio de Evolución de Sistemas Reproductivos en Angiospermas, Facultad de Biología, Universidad Michoacana de San Nicolás de Hidalgo, Morelia 58030, Mexico; eduardo.cuevas@umich.mx; 4 Centro de Investigaciones Biológicas del Noroeste S.C., La Paz 23096, Mexico; caricarix@gmail.com

**Keywords:** fly pollination, native bees, malformed fruits, *Mangifera indica*, pollination services, self-incompatibility

## Abstract

Adequate pollination of pollinator-dependent crops relies on the abundance and diversity of pollinators, and any temporal mismatch can lead to decreased productivity. Induced off-season flowering is widely used to anticipate the blooming time and to have a favorable market to generate greater economic income. However, the relationship between off-season flowering, effective pollination, and crop yield remains poorly understood. In this study, we compared pollinator and yield metrics of mango among its natural and off-season flowering across two years. We found that the composition, richness, and abundance of their effective pollinators varied across flowering seasons. Remarkably, blowflies were the floral visitors that deposited the highest number of pollen grains per visit and were the most important pollinators during the off-season, while honeybees and stingless bees were more important in the natural season. Mango yield was more positively related to the abundance of wild pollinators in both seasons than to honeybees. However, in both flowering seasons, mango trees suffered from pollen limitation and had a high incidence of malformed fruits. These findings highlight the important role of wild pollinators in maintaining and improving the mango yield and quality, mainly during the induced flowering season, improving the income to mango producers and increasing food security.

## 1. Introduction

The productivity of many crops worldwide is linked to the pollination services provided by animals [1,2,3] and depends on the abundance and diversity of pollinators present during their flowering [4]. For this reason, any mismatch between crop flowering and the presence of their pollinators can cause a deficient pollination service [4,5,6] and lower quantity and/or quality of fruits [2,7,8]. Thus, the use of particular agricultural practices, such as irrigation, changes in the date of sowing, and the use of chemical and hormonal treatments that alter the beginning of crop flowering [5,6,9,10], may affect the productivity of these crops. This is particularly important in tropical regions, where ~94% of crops depend on pollinators [11] and those agricultural practices are widely used [5,6,9]. Therefore, it is crucial to document whether these alterations in flowering seasons are associated with changes in pollinator composition, abundance and effectiveness, as well as the productivity of the crops they pollinate, to plan strategies that guarantee their pollination services and support food security [4].

One of the pollinator-dependent crops where chemical and hormonal treatments are commonly used to alter flowering is the mango (*Mangifera indica* L., Anacardiaceae [12]). This crop is of great economic importance in diverse tropical countries around the world [13,14], including Mexico, which is the largest producer in America and the main exporter of mango worldwide [15]. In particular, in southern Mexico, the ‘Ataulfo’ cultivar, one of their most economically important cultivars, naturally blooms between December and January (hereafter, “natural flowering” [16,17]). However, most producers apply nitrates and growth regulators, such as paclobutrazol [12], to stimulate flowering in the rainy season between September and November (hereafter “off-season flowering” [12]). This practice induces off-season flowering to advance the harvesting stage, so growers can supply markets with high demand and therefore obtain higher prices compared with the natural harvest season, generating greater economic benefits [18,19]. Nevertheless, since ‘Ataulfo’ is a varietally self-incompatible crop, a mismatch with compatible cultivars may increase the probability of ineffective pollination (i.e., pollen transfer from the same cultivar), increasing the production of nubbins (stenospermocarpic fruits). Nubbins are malformed fruits characterized by their smaller size and weight, which have little or no commercial value compared to commercial fruits [20], and which are associated with reductions in yield and economic income to producers [17,21].

In addition to the mismatch between crop flowering and pollinator activity and with compatible cultivars, other biotic and abiotic factors may also be associated with variation in crop yield and economic income. For instance, the off-season flowering coincides with the end of the rainy season (June–November), when environmental conditions such as temperature, precipitation, and wind speed can be adverse for mango flowers, floral visitors, and fruit set [21], as reported in other mango cultivars, apricots, and other wild species [22,23,24,25]. In particular, the cloudy climate and the rains during flowering cause serious damage to the mango flowers and cause their abortion [23]. Even considering these disadvantages, most producers choose to induce flowering because the demand for mangoes is very high and prices increase during the off-season [18].

Moreover, the response of floral visitors to climatic conditions can be specific [22]; whereas some insects are more abundant during the natural flowering season, others were more abundant during the off-season [5,6,26]. For example, honeybees (*Apis mellifera*), a widely managed species for crop pollination that contributes roughly half of the pollination services across 93 crops worldwide [27], were among the most abundant floral visitors of ‘Ataulfo’ mango during natural flowering [17,28]. However, they show greater sensitivity to rain, strong winds, and temperature changes compared to other groups of wild floral visitors, such as flies [22,29]. Therefore, the climatic conditions during the off-season flowering may influence the composition and abundance of floral visitors [23], potentially reducing pollination effectiveness (i.e., the product of abundance and pollen deposition on the stigma [30]), due to their lower activity during the rainy season. However, to our knowledge, no studies have evaluated the relationship between induced flowering, pollinators, and crop yield using a direct measure of the effectiveness of pollinators, such as the single-visit pollen deposition.

Reduced honeybee activity during the off-season flowering may not necessarily be unfavorable to mango production, as a high honeybee abundance has been negatively associated with the production of commercial fruits during natural flowering, mainly because they move less among trees [17] and because some flies have been found to be effective pollinators of this crop [17]. Nevertheless, the effect of this contrast between honeybees and wild insects during the off-season and the consequence on fruit production has not yet been evaluated.

The off-season flowering of ‘Ataulfo’ mango coincides with the flowering of most native plant species of the Mexican tropical dry forests (TDFs [31,32]), and given that focal crops are usually less attractive to floral visitors when floral resources in the landscape are high [33], it is expected that during the off-season, ‘Ataulfo’ flowers would experience high pollen limitation. In contrast, during the natural flowering season of ‘Ataulfo’ mango, floral resources on TDFs are scarce because the flowering peak of the native plants has already passed [31,32], which may increase the floral visitation to this crop [33].

The aim of this study is to compare the composition, richness, and abundance of the floral visitors of the ‘Ataulfo’ mango, their pollination effectiveness and contribution, the impact of the abundance of honeybees and wild floral visitors on the yield of commercial fruits, as well as the quantity and quality of the commercial fruits, the incidence of nubbins, and the pollen limitation of mango orchards in the natural and in the off-season flowering during two consecutive years.

Five predictions with their respective hypotheses were proposed: (1) the composition of floral visitors will differ between both flowering seasons given that the composition of floral visitors is associated with seasonality; (2) the abundance of floral visitors and their pollination effectiveness will be lower during the off-season flowering because floral resources of the native vegetation are abundant and the climatic conditions will be adverse, compared to the natural flowering season; (3) pollen limitation and the incidence of nubbins will be higher during the off-season flowering, while the production of commercial fruits and yield will be lower given the lower abundance of floral visitors compared to the natural flowering; (4) the importance of honeybees as pollinators will be lower during the off-season flowering, as honeybees are more susceptible to the adverse environmental conditions of this season than other wild insects such as flies; and (5) wild insects’ abundance will be positively related to the mango yield in both flowering seasons, given that honeybees move less among trees compared to wild pollinators.

## 2. Results

### 2.1. Composition of Floral Visitors

**Figure 1:**
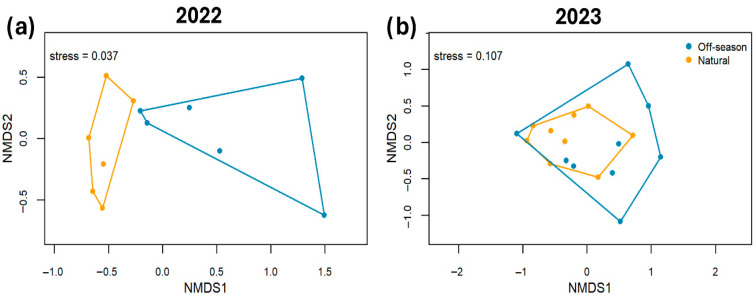
A non-metric multidimensional scaling (NMDS) of the floral visitor communities of the ‘Ataulfo’ mango during the off-season and natural flowering in (**a**) 2022 and (**b**) 2023 in the Costa Grande region, Guerrero, Mexico. Analyses were conducted separately for each year.

In total, 8609 insects were observed visiting the ‘Ataulfo’ mango flowers in 12 orchards in 2022 (3622 visitors) and in 18 orchards in 2023 (4987 visitors, Table S1). The insects belonged to 49 different species or morphospecies (Table S1). In general, we found that the composition of the floral visitors was different between the off-season and natural flowering in 2022 (*R*^2^ = 0.33, *p* = 0.002, stress = 0.037, Figure 1a), but not in 2023 (*R*^2^ = 0.09, *p* = 0.136, stress = 0.107, Figure 1b). In 2022, indicator species analyses identified the blowfly *Compsomyiops* sp. 1 as the only species significantly associated with the off-season flowering (IndVal = 0.93, *p* = 0.035), whereas no species were significantly associated with the natural flowering season. In 2023, no species showed significant associations with either flowering season.

### 2.2. Richness and Abundance of Floral Visitors

In 2022, the number of floral visitor species did not differ significantly between both flowering seasons (13.8 vs. 12.5; *χ*^2^ = 1.01, df = 1, *p* = 0.31). In contrast, in 2023, the number of species recorded during natural flowering was 34% higher than during the off-season flowering (17.1 vs. 12.8; *χ*^2^ = 6.10, df = 1, *p* = 0.01, Figure S1).

**Figure 2:**
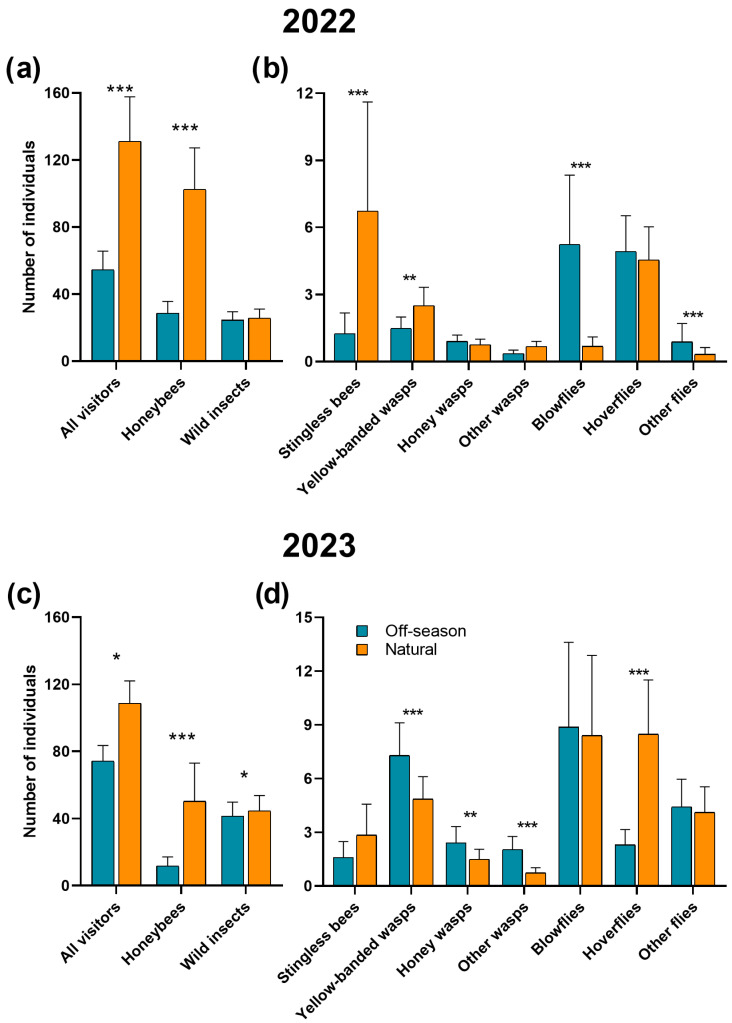
Mean abundance of floral visitors in ‘Ataulfo’ mango during the off-season and natural flowering in (**a**,**b**) 2022 and in (**c**,**d**) 2023, by all floral visitors, honeybees, and wild insects (**a**,**c**) and by the seven subgroups of wild insects (**b**,**d**) in the Costa Grande region, Guerrero, Mexico. Averages ± SE are shown. The asterisks above bars indicate significant differences among flowering seasons according to Tukey’s test (*: *p* < 0.05, **: *p* < 0.01, ***: *p* < 0.001). Analyses were conducted separately for each year.

In 2022, during the off-season flowering, the most abundant floral visitors were honeybees (54%), followed by blowflies (20%) and hoverflies (11%), while in the natural flowering season, the honeybees (80%), stingless bees (20%), and hoverflies (4%) were the most common (Figure 2a,b). In 2023, the most abundant floral visitors during the off-season flowering were again the honeybees (37%), followed by blowflies (26%) and yellow-banded wasps (12%), while in the natural flowering, the honeybees (53%), blowflies (17%), and hoverflies (11%) were the most common (Figure 2c,d).

The abundance of all floral visitors was significantly greater during the natural compared to the off-season flowering in both years (2022: *χ*^2^ = 636.78, df = 1, *p* < 0.001, Figure 2a; 2023: *χ*^2^ = 5.78, df = 1, *p* = 0.01, Figure 2c). Specifically, the abundance of honeybees was greater during the natural than during the off-season flowering in both years (2022: *χ*^2^ = 887.9, df = 1, *p* < 0.001, Figure 2a; 2023: *χ*^2^ = 15.63, df = 1, *p* < 0.001, Figure 2c), while the abundance of wild insects in 2023 was greater during the natural than during the off-season flowering (*χ*^2^ = 3.89, df = 1, *p* = 0.048, Figure 2c), but did not differ in 2022 (*χ*^2^ = 0.145, df = 1, *p =* 0.702, Figure 2a).

In 2022, the abundance of stingless bees and yellow-banded wasps was greater during the natural flowering compared to the off-season flowering (*χ*^2^ = 161.24, df = 1, *p* < 0.001; *χ*^2^ = 6.01, df = 1, *p* = 0.014, respectively). In contrast, blowflies and other flies were more abundant during the off-season flowering (*χ*^2^ = 164.36, df = 1, *p* < 0.001; *χ*^2^ = 14.90, df = 1, *p* < 0.001, respectively). The rest of the groups show similar abundances among both flowering seasons (*χ*^2^ < 1.83, df = 1, *p* > 0.05 in all cases, Figure 2b).

In 2023, hoverflies were observed at higher abundance during the natural compared to the off-season flowering (*χ*^2^ = 144.67, df = 1, *p* < 0.001). In contrast, the yellow-banded wasps, honey wasps, and the other wasps were more abundant during the off-season flowering (*χ*^2^ = 16.31, df = 1, *p* < 0.001; *χ*^2^ = 9.11, df = 1, *p* = 0.002; *χ*^2^ = 25.86, df = 1, *p* < 0.001, respectively). The remaining floral visitors showed similar abundances across both flowering seasons (*χ*^2^ < 0.80, df = 1, *p* > 0.05 in all cases, Figure 2d).

### 2.3. Pollinator Effectiveness and Relative Contribution of Each Pollinator

**Figure 3:**
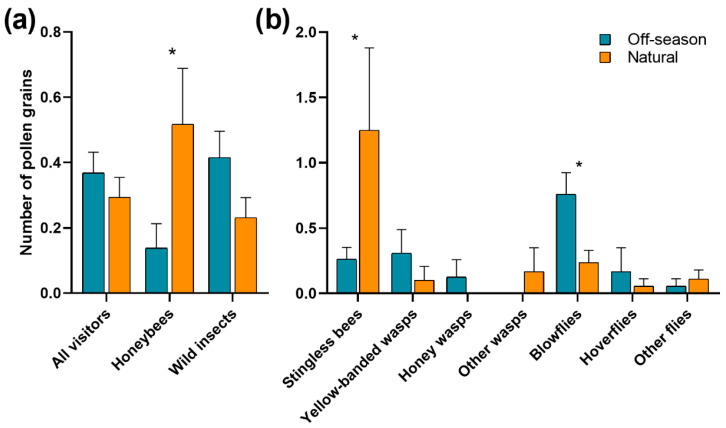
Mean pollen grain deposition of different floral visitors of the mango ‘Ataulfo’ during the off-season and natural flowering in 2023 by (**a**) all floral visitors, honeybees, and wild insects and by (**b**) the seven subgroups of wild insects in the Costa Grande region, Guerrero, Mexico. Averages ± SE are shown. Asterisks above bars indicate significant differences among flowering seasons according to Tukey’s test (*: *p* < 0.05).

The number of pollen grains deposited on stigmas by all floral visitors in 2023 varied from 0 to 6 grains both in off-season (n = 171 stigmas, Table S2) and natural flowering (n = 133 stigmas, Table S2). Considering all the floral visitors of ‘Ataulfo’, there was no difference in the number of pollen grains deposited on stigmas between flowering seasons (*χ*^2^ = 0.70, df = 1, *p* = 0.40). However, significant differences were observed in the deposition of pollen between the different groups of floral visitors (*χ*^2^ = 24.06, df = 7, *p* = 0.001, Figure 3a). The blowflies (0.56 ± 0.11; mean ± SE), stingless bees (0.42 ± 0.12) and honeybees (0.33 ± 0.09) deposited a greater number of pollen grains than the rest of the floral visitors (<0.22). A significant interaction between flowering season and pollinator group was detected (*χ*^2^ = 21.38, df = 7, *p* = 0.003, Figure 3). Specifically, the honeybees and the stingless bees deposited more pollen per visit during the natural than during the off-season flowering, while the blowflies deposited more pollen per visit during the off-season than during the natural flowering (Figure 3).

**Figure 4:**
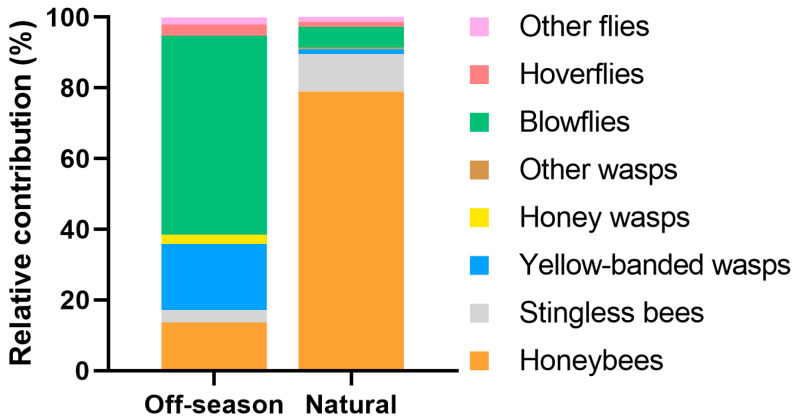
Relative contribution (%) of each floral visitor to the effective pollination of ‘Ataulfo’ during the off-season and natural flowering of 2023 in the Costa Grande region, Guerrero, Mexico.

The blowflies were the most effective pollinators during the off-season flowering, followed by yellow-banded wasps and honeybees (Table 1). During the natural flowering, the honeybees were by far the most effective pollinators, followed by the stingless bees. In general, the contribution of wild pollinators to the effective pollination of ‘Ataulfo’ was greater during the off-season flowering (86%) than during the natural flowering (21%; Figure 4).

### 2.4. Pollen Limitation

**Figure 5:**
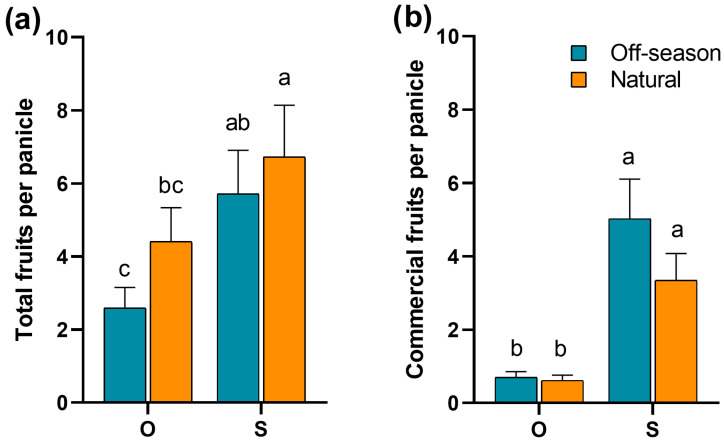
Average number (±SE) of (**a**) total fruits (commercial plus malformed fruits) (**b**) and commercial fruits per panicle of mango ‘Ataulfo’ produced under open (O) and supplementary pollination (S) with ‘Haden’ pollen in 2023 in the Costa Grande region, Guerrero, Mexico. Different letters above bars indicate significant differences among pollination treatment and flowering seasons according to Tukey’s test (*p* < 0.05).

There was evidence of strong pollen limitation (*χ*^2^ = 83.99, df = 2, *p* < 0.001), since fruit production under the supplemental pollination treatment was almost twice as high (6.19 ± 0.94) compared to the open pollination treatment (3.43 ± 0.53) in both flowering seasons (*χ*^2^ = 8.62, df = 1, *p* = 0.003, Figure 5a). Considering only the production of commercial fruits per panicle, significant differences were also found among treatments (*χ*^2^ = 361.31, df = 1, *p* < 0.001). Specifically, the supplementary pollination treatment (4.12 ± 0.71) produced significantly more commercial fruits than the open pollination treatment (0.65 ± 0.12, Figure 5b). Finally, there was no difference in the total fruit production (*χ*^2^ = 0.22, df = 1, *p* = 0.64, Figure 5a) or the production of commercial fruits (*χ*^2^ = 0.06, df = 1, *p* = 0.80, Figure 5b) among seasons within the same treatment. Although pollen limitation did not differ between seasons, data showed considerable variation in pollen limitation among orchards (Table S3).

### 2.5. Commercial Fruit Production and Malformed Fruit Incidence

**Figure 6:**
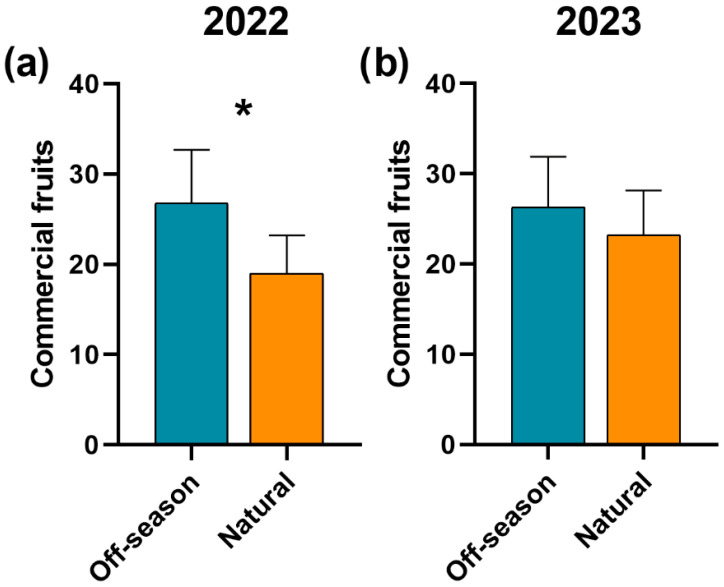
Average production (±SE) of commercial fruits in mango ‘Ataulfo’ during two flowering seasons in (**a**) 2022 and in (**b**) 2023 in the Costa Grande region, Guerrero, Mexico. Asterisks above bars indicate significant differences among flowering seasons according to *χ*^2^ tests followed by post hoc comparisons (*: *p* < 0.05). Analyses were conducted separately for each year.

The production of commercial fruits was greater during the off-season than in the natural flowering in 2022 (*χ*^2^ = 4.35, df = 1, *p* = 0.03, Figure 6a), though, in 2023, it was similar between seasons (*χ*^2^ = 0.31, df = 1, *p* = 0.57, Figure 6b). On the other hand, the incidence of nubbins was similar between both flowering seasons in 2022 (off-season: 0.42 ± 0.14, natural: 0.40 ± 0.13; *χ*^2^ = 0.06, df = 1, *p* = 0.79) and in 2023 (off-season: 0.24 ± 0.12, natural: 0.31 ± 0.14; *χ*^2^ = 0.52, df = 1, *p* = 0.46).

### 2.6. Commercial Fruit Quality and Yield

**Figure 7:**
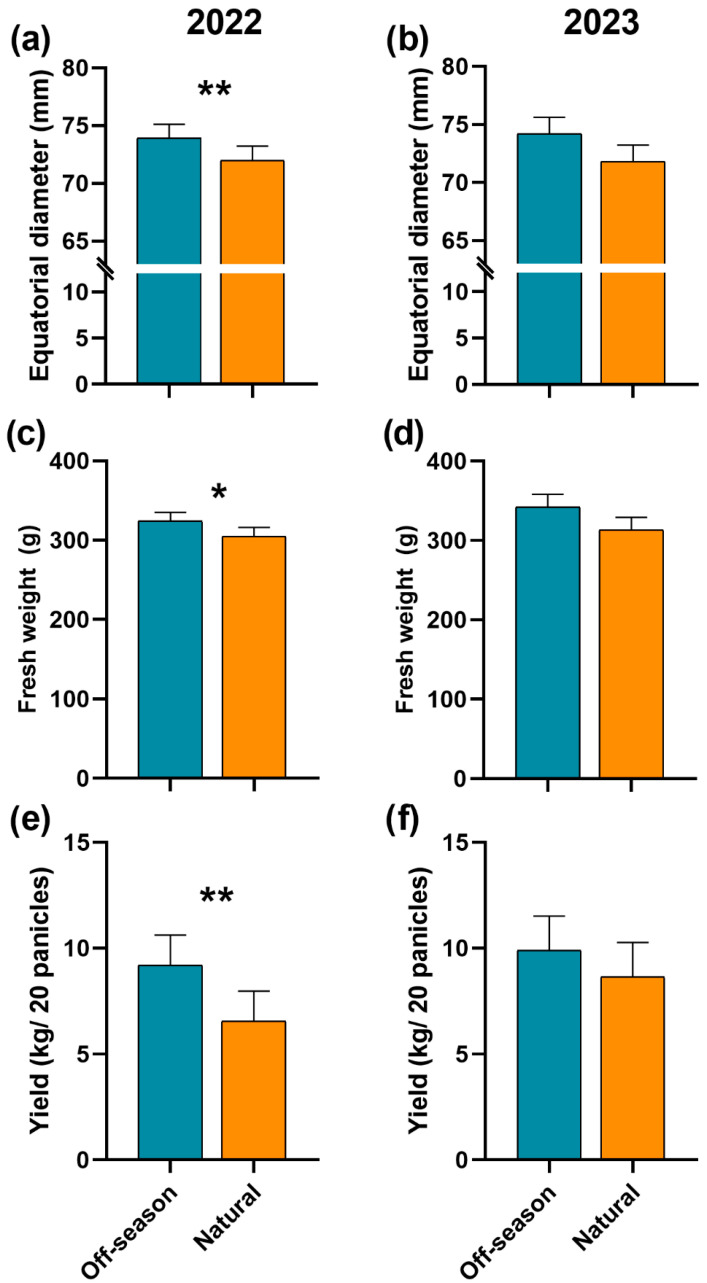
(**a**,**b**) Average size, (**c**,**d**) weight and (**e**,**f**) yield (±SE) of commercial fruits of mango ‘Ataulfo’ produced during two flowering seasons in (**a**,**c**,**e**) 2022 and (**b**,**d**,**f**) 2023 in the Costa Grande region, Guerrero, Mexico. Asterisks above bars indicate significant differences among flowering seasons according to *χ*^2^ tests followed by post hoc comparisons (*: *p* < 0.05, **: *p* < 0.01). Analyses were conducted separately for each year.

The polar diameter of the commercial fruits was similar between the flowering seasons in both years (2022: *χ*^2^ = 2.14, df = 1, *p* = 0.14; 2023: *χ*^2^ = 1.09, df = 1, *p* = 0.29). In contrast, the fruits presented a larger equatorial diameter (*χ*^2^ = 7.06, df = 1, *p* = 0.007, Figure 7a) and were heavier than those produced during the natural flowering (*χ*^2^ = 5.92, df = 1, *p* = 0.01, Figure 7c) during the off-season flowering in 2022. However, in 2023, no differences were found in the equatorial diameter (*χ*^2^ = 1.60, df = 1, *p* = 0.20, Figure 7b) or fresh weight (*χ*^2^ = 1.79, df = 1, *p =* 0.18, Figure 7d) among flowering seasons.

The yield of commercial fruits of 20 panicles was greater during the off-season compared with the natural flowering in 2022 (*χ*^2^ = 7.82, df = 1, *p* = 0.005, Figure 7e), though in 2023, it was similar to the off-season (*χ*^2^ = 0.76, df = 1, *p* = 0.38, Figure 7f).

### 2.7. Association Between Floral Visitors’ Abundance and Mango Yield

**Figure 8:**
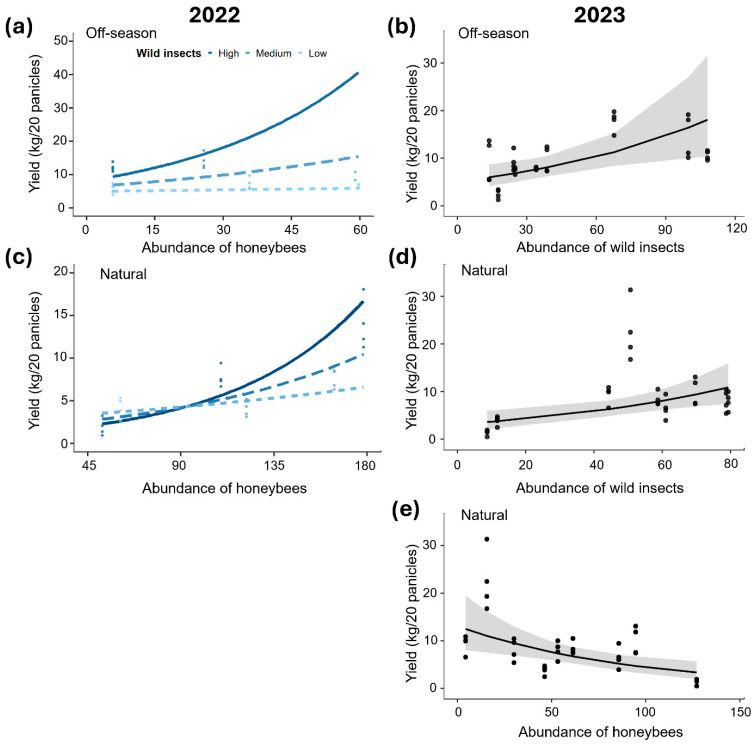
Relationships for the yield of ‘Ataulfo’ mango with the abundance of wild insects and the abundance of honeybees during the off-season and natural flowering season of 2022 and 2023 in the Costa Grande region, Guerrero, Mexico. Interaction between honeybee abundance and wild insect abundance in relation to yield (**a**) during the off-season in 2022 and (**c**) the natural flowering season in 2022. Relationships between wild insect abundance and yield (**b**) during the off-season in 2023 and (**d**) the natural flowering season in 2023, and (**e**) between honeybee abundance and yield during the natural flowering season in 2023. In the interaction plots (**a**,**c**), the effects of increased honeybee abundance are plotted for different wild insect abundances: high abundance (mean + 1 SD; solid blue line), medium abundance (mean; light blue dashed line), and low abundance (mean − 1 SD; light blue dotted line). The solid lines and gray bands indicate the GLMM fit and 95% confidence interval, respectively. The dots represent the estimated results for each orchard. Analyses were conducted separately for each year.

The relationship between floral visitors’ abundance and commercial fruit yield of mango ‘Ataulfo’ varied among years and flowering seasons. In 2022, during the off-season flowering, mango yield was positively related to the abundance of wild insects (*β* = 0.63 ± 0.07, *p* < 0.001) and honeybees (*β* = 0.34 ± 0.06, *p* < 0.001). During the natural flowering season, the yield was positively related to honeybee abundance (*β* = 0.50 ± 0.09, *p* < 0.001), but not to wild insect abundance (*β* = 0.12 ± 0.09, *p* = 0.22). During both flowering seasons, a positive interaction was observed between the abundance of honeybees and wild insects in relation to mango yield (off-season: *β* = 0.27 ± 0.06, *p* < 0.001; natural: *β* = 0.26 ± 0.09, *p* = 0.004). This interaction indicates that the yield was significantly greater when both groups of floral visitors were highly abundant (Figure 8a,c).

In 2023, the yield was only positively related to wild insect abundance during the off-season flowering (*β* = 0.40 ± 0.14, *p* = 0.005, Figure 8b) and during the natural flowering season (*β* = 0.39 ± 0.13, *p* = 0.003, Figure 8d). In contrast, during the natural flowering season, the yield showed a negative relationship with honeybee abundance (*β* = −0.41 ± 0.13, *p* = 0.002, Figure 8e), whereas no significant association was detected during the off-season flowering (*β* = 0.21 ± 0.14, *p* = 0.143).

## 3. Discussion

Our study offers the first comparative evaluation of the dynamics of the effective pollinators of the ‘Ataulfo’ mango between two contrasting flowering seasons, one natural and one altered (off-season), for two years. As predicted, our results indicate that an alteration in floral phenology was associated with changes in the composition of floral visitors, and that their abundances were higher during the natural flowering season, though the effectiveness was not always higher in the natural season for all floral visitors. Contrary to our predictions, pollen limitation and the incidence of nubbins were similar during both seasons, while the production of commercial fruits was higher during the off-season in one year. As suspected, honeybees and flies were more important as effective pollinators during the natural and off-season flowerings, respectively. Finally, as predicted, the abundance of wild insects was positively related to mango yield in both flowering seasons, with the exception of the natural season in one year.

### 3.1. Temporal Variation in the Composition, Richness and Abundance of Floral Visitors

Even when honeybees were a very common floral visitor, a great diversity of wild insects visiting the mango flowers (i.e., 47 species) was also recorded, similar to previous studies performed in this region also with mango [17,21,28]. In 2022, differences in the composition of floral visitors between the off-season and natural flowering seasons could be related to differences in the precipitation regime, since rainfall was only recorded during the off-season flowering, whereas in 2023, rainfall was also recorded during the natural flowering season (Figure S2a). On the other hand, the differences observed in richness between seasons could be caused by physiological and behavioral responses of insects to environmental temperature, which could have influenced the availability and diversity of pollinators [22]. For instance, in 2022, when temperatures were similar in September and December, species richness was similar between flowering seasons; whereas, in 2023, when temperatures in September were higher than in December, species richness was greater during the natural flowering season (Figure S2b). Environmental temperature variation has also been shown to influence the richness of floral visitors. For example, the richness of butterflies [34] and fly communities [35,36] has been found to increase in the warmer season compared to the cooler season [34,35,36].

The total abundance of floral visitors was consistently greater during the natural flowering in both years, driven mainly by a greater presence of honeybees. This pattern has also been observed in other agricultural systems, as in longan (*Dimocarpus longan*), where the off-season flowering mainly attracts dipterans, while wild bees and honeybees were more abundant in the natural flowering [6]. Honeybees are highly sensitive to environmental conditions such as rain, strong winds, and temperature fluctuations, which can limit their foraging activity compared to many wild insects that tend to be more tolerant to these conditions [22,29,37]. Indeed, in 2022, during the off-season flowering, blowflies and other flies showed a greater abundance than during the natural flowering, possibly due to their tolerance to higher humidity and moderate temperatures [37], as well as the availability of microhabitats for reproduction, such as decomposing organic matter. Consistent with this pattern, the only indicator species associated with this flowering season was the blowfly *Compsomyiops* sp. 1. On the other hand, in 2023, wasps were more abundant during the off-season flowering. These findings accord with other studies suggesting that the composition and abundance of floral visitors respond differently to environmental and seasonal changes [5,6,26,36]. Long-term research is needed to gain a deeper understanding of how environmental conditions influence the pollination services provided by different groups of pollinators in mango and other crops.

The availability of alternative floral resources between seasons may also affect the composition, richness, and abundance of floral visitors. During the off-season flowering of the ‘Ataulfo’ mango, many native plants overlap in their flowering season, which could generate a high supply of floral resources in the environment [31,32]. It has been found that the high availability of alternative resources reduces the fidelity of pollinators to focal crops [33,38,39]. This could explain the higher abundance of all floral visitors and particular groups in the natural flowering in at least one year. In social bees such as *Apis mellifera*, foraging decisions are influenced by the profitability of floral resources and colony-level recruitment dynamics [40,41]. Thus, lower visitation by honeybees during the induced off-season may reflect shifts in foraging allocation rather than a reduced pollination effectivity [41]. In addition, seasonal fluctuations in honeybee abundance may partly reflect apicultural practices, such as colony placement, hive density, or the seasonal movement of managed colonies, rather than a response to floral phenology [42]. However, information on hive density or beekeeping management in the surrounding landscape was not recorded during this study and should be evaluated in the future, as well as the influence of the supply of floral resources in and around mango orchards on the temporal dynamics of floral visitors.

It is also important to consider that pollinator abundance can be influenced by landscape context. A previous study with ‘Ataulfo’ in the same region has shown that visitation rates decline with increasing distance from remnants of TDF, which may act as an important source of pollinators in agricultural landscapes [28]. Even when our experimental design (consisted of orchard pairs located in proximity) helped to reduce local environmental variation, differences in landscape composition among orchard pairs, including variation in distance to forest remnants or the amount of surrounding natural vegetation, may still influence the spatial variation observed in pollinator communities.

### 3.2. Temporal Variation in the Contribution of Pollinators

The specific contribution to ‘Ataulfo’ pollination during the off- and natural flowering seasons across both years might be explained by differences in the abundance and in the capacity of each pollinator to deposit pollen on conspecific stigmas [17,43]. While insect abundances may respond, at least partially, to environmental cues as explained above, differences in per-visit efficiency may be related to factors such as interspecific competition, foraging behavior, and body morphology, which may influence pollen transport and contact with the reproductive structures of mango flowers [6,44]. Possibly due to the reduced abundance of honeybees during the off-season flowering, the blowflies could have stayed longer in each flower [45], increasing the probability of deposition of pollen [46]. The behavior of pollinators may also change if the concentration and production of nectar and the availability of pollen are affected by variation in abiotic factors such as temperature or humidity [47,48], thus influencing their effectiveness [6]. For example, if a plant has more diluted nectar after rain, it could favor cross-pollination, since it would force pollinators to visit more flowers to satisfy their energy needs [47]. However, floral rewards were not quantified in the present study. Future studies are needed to evaluate if mango floral rewards change during the different seasons of flowering.

### 3.3. Strong Pollen Limitation and High Nubbins Incidence in Both Flowering Seasons

The strong pollen limitation observed in 2023 (the only year in which it was estimated) in both flowering seasons suggests that the pollination services available in the studied mango orchards were insufficient to maximize its fruit production. These results are consistent with a recent study showing that globally, 28–61% of crops show pollen limitation due to a lack of pollinators [49]. Although pollen limitation was similar among flowering seasons, it is important to note that pollen limitation varied among orchards (Table S3). When only commercial fruit production was considered, pollen limitation varied from 50 to 100% among orchards, suggesting that the efficiency of pollination services in mango varies greatly spatially. Such variation has also been reported in other fruit tree crops, such as macadamia, apple, and pear [50,51,52]. This has been attributed to differences in pollinator abundance, orchard structure, or local management [50,51,53]. Given that most of the orchards (eight out of nine) were managed by the same family and pollen limitation was similar between seasons in our study, we consider that most of the spatial variation in pollen limitation is mainly due to differences in pollinator availability and diversity, rather than to differences in local management practices and orchard structure (e.g., use of insecticides, density and location of pollinizer trees, that is, compatible cultivars such as ‘Haden’ [16]). However, it is also important to consider that the relatively low density and scattered distribution of pollinizer trees may have limited pollen flow and contribute to the observed pollen limitation. We suggest exploring this factor in future studies.

On the other hand, it is important to mention that in the open pollination treatment, we find a high production of nubbins, which suggests that fruit production was limited by inadequate pollination. In the case of ‘Ataulfo’, it is the result of an insufficient supply of compatible pollen during pollination, since the formation of nubbins in this cultivar has been associated with pollination within the same cultivar due to the presence of varietal self-incompatibility [16,54]. Furthermore, this could have been exacerbated considering that the main floral visitors to mango were honeybees, which generally feed within the same tree [17] and move along rows of trees rather than between rows [17,55], favoring pollination within the same cultivar and, consequently, a high incidence of nubbins or fruit abortion.

The incidence of nubbins, which was similar between seasons, is considered one of the main factors that limits the productivity of ‘Ataulfo’ [16,28]. Even when, in recent years, the exploitation of these malformed fruits has been promoted [56,57], their commercial value is very low and causes large economic losses ~90% of production in some regions [58,59]. Therefore, in addition to promoting strategies to strengthen pollinator communities, mango producers should include co-flowering cultivars compatible with ‘Ataulfo’ in the same orchard to increase the availability of compatible pollen [21], reduce pollen limitation, and decrease nubbins incidence.

### 3.4. Wild Pollinators Are Crucial for the Increase in the Quantity and Quality of Fruits

Honeybees are currently one of the most widely used pollinators for crop pollination worldwide [60,61]. Although it is believed that a greater quantity of honeybees translates into greater pollination and production of fruits and seeds [60,62], several studies indicate that these insects are not necessarily the most effective pollinators [27,63,64], especially in species with some degree of self-incompatibility [17,64], given that they increase the deposition of incompatible pollen due to their food-seeking pattern [29,65]. Moreover, recent evidence has shown that an excess of visits by honeybees can lead to a reduction in fruit production [62] and a negative impact on the wealth and abundance of wild pollinators by competing for floral resources in orchards [46,62]. Similarly, in this study, we found that in one year, the increase in the visits of honeybees during the natural flowering decreased the production of commercial fruits. In contrast, wild pollinators’ abundance usually shows a positive relationship with mango yield, possibly due to a more favorable behavior of these insects, which move more often between different trees, promoting better cross-pollination [17,29,65]. These results are consistent with recent studies that also found a positive relationship between the abundance of wild insects and the productivity of ‘Ataulfo’ mango [17,28].

We also found that when the abundance of wild insects was low, the mango yield did not increase with an increase in the abundance of honeybees, but there was a positive relationship when the abundance of wild insects increased simultaneously, which suggests that wild insects could facilitate the pollination service performed by honeybees [29,65]. Indeed, in diverse crops, it has been documented that the presence of wild insects can indirectly improve the efficiency of honeybees by increasing their rate of visits, the probability of changing rows, and the effectiveness of each visit [29,65]. Moreover, our results suggest that, in particular, blowflies play an important role in mango pollination, complementing the activity of other pollinator groups such as honeybees and wasps across flowering seasons, contributing to fruit production and quality, as has been shown in other studies (e.g., [17,66]). In sweet cherries, for example, interactions between wild and managed pollinators have been shown to enhance fruit set [67].

Our results also show that not only fruit quantity varies among seasons but also fruit quality. Commercial fruits produced during the off-season were of better quality, presenting a significantly larger equatorial diameter and fresh weight compared to those obtained in the natural flowering, which has direct implications in its commercial value, acceptance in the market, and economic income for producers [16,21]. A better quality of fruits has been associated with better pollination, characterized by a greater amount of compatible pollen [63].

The greater contribution of wild pollinators, mainly during the off-season, suggests that different functional groups of pollinators play a complementary role to ensure the pollination service over time [37]. This complementarity is very important to maintain the productivity of crops [37], especially on those that present multiple seasons of flowering. In fact, in other agricultural systems, it has been documented that the presence of a greater diversity of pollinators with different climate sensitivities strengthens the resilience of the pollination service in the face of environmental variability [68]. Together, these findings support the growing evidence that the functional diversity of pollinators not only ensures the pollination service but also enhances its effectiveness through positive interactions between species [29,65,67].

Even though pollination is considered one of the main limiting factors in the production of fruits in tree crops [69,70], other factors such as the characteristics of the tree, the type of management, the soil properties, or the climatic conditions can also influence the production and retention of fruits in orchards after successful pollination [69,70]. For instance, it has been documented that water stress negatively affects the production of fruits in crops such as almonds and mangosteens [71,72]. Nevertheless, because all the orchards had an irrigation system, and because, in the drier year (i.e., 2022, Figure S2), there was higher production of fruits and yield, we exclude water availability as a main limiting factor for fruit production in our study area.

All these results suggest that a higher quantity and quality of commercial fruits across seasons is due to the interaction between environmental conditions and adequate pollination by flies, bees, and wasps. Moreover, the positive relationship between the abundance of wild floral visitors and the yield of ‘Ataulfo’ mango suggests that wild pollinators play an important role in mango productivity during flowering seasons when environmental conditions are less favorable for other potential pollinators, such as honeybees, contributing to increased and stabilized crop yields, particularly when honeybees are absent or less active [2,63].

## 4. Materials and Methods

### 4.1. Study Area

**Figure 9:**
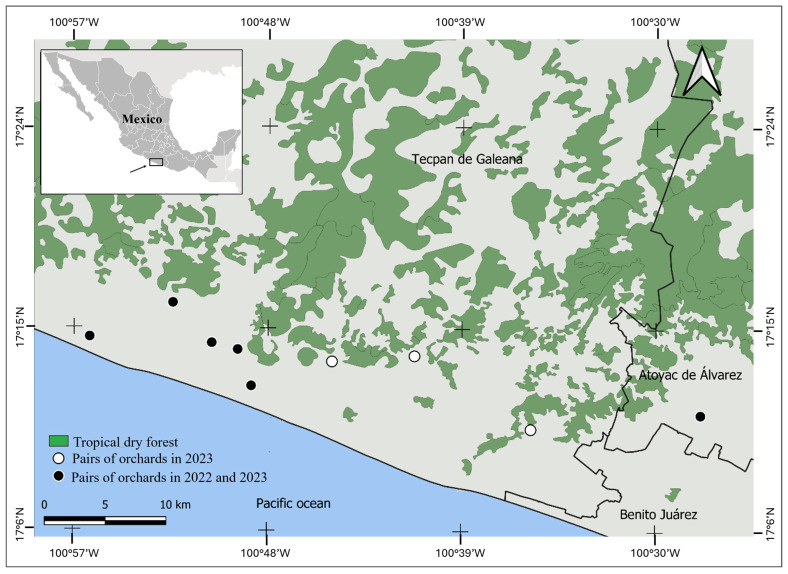
Geographical location of nine pairs of orchards of mango ‘Ataulfo’ selected for this study during 2022 and 2023 in the Costa Grande region, Guerrero, Mexico. Municipality names are shown in Spanish.

This study was carried out in the Costa Grande region of the state of Guerrero, in southern Mexico (Figure 9), one of the major mango-produced regions in Mexico [73]. The main native vegetation is TDF surrounded by crops of mango, coconut, and banana [74], as well as subsistence agriculture (maize and beans) and cattle ranches. The climate of the region is warm subhumid (Aw), with an average annual precipitation of 1100 mm. The rainy season is from June to November (total precipitation ≈ 950 mm), and a dry season from December to May (total precipitation < 70 mm). The average annual temperature is 26 °C, with a maximum of 32 °C in April–May and a minimum of 18 °C in December-January [75]. Monthly temperature and precipitation data during the flowering seasons of both years were compiled to analyze possible relationships with the variation in pollinator and fruit production data. This information was obtained from a meteorological station of the Mexican National Meteorological Service located within the study area (Figure S2).

### 4.2. Study Species

The mango is an andromonoecious tree (i.e., it has male and hermaphrodite flowers on the same plant) with panicle-shaped inflorescences that can vary from a few hundred flowers to several thousand [76,77], while the proportion of hermaphrodite and male flowers depends on the cultivar and management practices [76]. The anthesis is diurnal, and flowers are visited by flies, bees, wasps, and beetles [17,28,76,77,78]. ‘Ataulfo’ is a varietally self-incompatible cultivar that requires cross-pollination with particular cultivars to increase crop yield [16,54,79]. Thus, only mixed orchards were selected, with at least one cultivar ‘Haden’ tree as a pollen source (pollinizer), which has been found to be an effective pollen donor for ‘Ataulfo’ [16]. In most orchards, ‘Haden’ trees were not arranged in a specific spatial pattern and were instead scattered within the orchard, typically at low densities (approximately 1–4 trees per hectare). Only in one orchard (i.e., Cuauhtémoc) pollinizer trees were planted along the borders of blocks of ‘Ataulfo’ trees.

### 4.3. Experimental Design

We selected six and nine orchard pairs in 2022 and 2023, respectively. Each pair consisted of one off-season (blooming from mid-October to mid-November) and one natural flowering orchard (blooming from early December to early January). The six pairs sampled in 2022 were included in 2023. Orchards of each pair were located in proximity (<100 m) to minimize variation in soil characteristics and local environmental conditions. The average distance between a pair of orchards was 19.0 ± 2.1 km (mean ± SE, range: 2.2–50.5 km, Table S4). In the majority of pairs (8 of 9), both orchards were owned by the same family, which contributed to reduced variation in management practices. In the other pair of orchards, the producers discussed their management practices with us, and their practices were also similar. The average age of trees in all orchards was 16 ± 0.9 years, and they were grown under similar conventional management practices (e.g., use of pesticides and synthetic fertilizers). All selected orchards had an average size of 3.8 ± 0.5 ha, planted under a square planting system, with irrigation through microsprinklers, with no managed honeybee hives introduced within the orchards during this study.

### 4.4. Composition, Richness and Abundance of Floral Visitors

Floral visitors were observed in three transects (60 × 2 m) of each orchard to estimate how the flowering season affects the floral visitation, its richness, composition, and abundance (e.g., [80]). Each transect, located next to a row of ‘Ataulfo’ mango trees, was walked slowly for 10 min three times a day, during the peak activity of floral visitors (i.e., 10:00, 13:00, and 16:00 h [28]). Each orchard was sampled one day during the time of maximum flowering, avoiding windy or rainy days. On each transect, all the insects visiting the mango flowers were recorded and collected to be identified at the lowest possible taxonomic level. The floral visitors were grouped into (1) honeybees (*A. mellifera*), (2) stingless bees (e.g., *Frieseomelitta nigra*), (3) yellow-banded wasps (i.e., *Polybia occidentalis*), (4) honey wasps (i.e., *Brachygastra azteca*), (5) other wasps (i.e., *Polistes* spp.), (6) blowflies (i.e., family Calliphoridae), (7) hoverflies (i.e., family Syrphidae), and (8) other flies (flies from the families Muscidae, Sarcophagidae, Tabanidae, and Tachinidae).

### 4.5. Pollinator Effectiveness and Relative Contribution of Each Pollinator

The pollinator effectiveness (PE) was estimated for 2023 as the product of the abundance of floral visitors and the number of pollen grains deposited on the stigma after a single visit for each flowering season. To calculate the latter, six panicles were chosen from each of four trees selected at random in each orchard (24 panicles per orchard), and they were enclosed in bags of fine mesh (40 × 30 cm; mesh size ~0.5 mm) to exclude floral visitors. On the following day, the bags were carefully removed, and the newly opened flowers in each panicle (approximately 10–15 flowers) were identified and observed until they received a floral visitor. This procedure was carried out at 10:00 h, when the dehiscence of the anthers began [21] and the receptivity of the stigma is optimal [54]. After a visit, the stigma was removed and placed on a microscope slide in the field with a gelatin-fuchsin cube (~3 × 3 mm), which was heated until the gel melted [81]. The prepared slides were stored and transported to the laboratory where pollen grains were counted on the same day with an optical microscope, and classified as conspecific or heterospecific pollen. Only conspecific pollen grains were detected on all the analyzed stigmas, that is, no heterospecific pollen was observed in any sample. Only unknown floral visitors (see previous section) were collected, mounted and stored for taxonomic identification. The relative contribution (RC) of each floral visitor was calculated as the proportion of PE of each species relative to the total sum of the PE of all species and shown as a percentage.

### 4.6. Pollen Limitation

To estimate pollen limitation, in 2023, four ‘Ataulfo’ mango trees located near a ‘Haden’ tree (7–10 m) were selected in each orchard, and six panicles with similar characteristics, which were located at the same height of the soil and similar in size and development status [16,28], were assigned to one of the following treatments (three panicles per treatment per tree): (1) open pollination—flowers were exposed to all floral visitors from anthesis to flower closure, and (2) supplementary pollination—flowers were manually pollinated touching the ‘Ataulfo’ stigma with pollen from dehisced anthers of ‘Haden’ cultivar and left exposed to all floral visitors. The number of developing fruits per panicle of each treatment was quantified 15 days after the establishment of the treatments (i.e., initial pruning) to minimize the effect of the tree’s load capacity [82], since the rate of fruit abscission is very high in this crop [21]. Pollen limitation was calculated as PL = 1 − OP/SP, where PL is pollen limitation, OP is the number of fruits resulting from open pollination, and SP is the number of fruits from the supplementary pollination treatment. Pollen limitation was estimated for each tree, and can vary from zero (without pollen limitation) to one (complete pollen limitation [83]).

### 4.7. Quantity and Quality of Fruits and Yield

To compare mango fruit quantity and quality across flowering season, we estimated the number, size, and weight of commercial fruits, the nubbins incidence, and the yield in 20 panicles per tree with similar characteristics in four trees selected at random, which were located on the same transects used for floral visitor observations. The total number of commercial fruits and the nubbins incidence (i.e., number of malformed fruits/total fruits per panicle) for each tree were counted 80 days after the flowering season, approximately two weeks before the commercial harvest period [28]. To compare the quality of commercial fruits in both flowering seasons, the polar and equatorial diameters of five commercial fruits selected at random were calculated using a digital caliper (Mitutoyo Corp., Model CD-8”ASXL, Kanagawa, Japan, accuracy 0.01 mm), while the fresh weight was estimated using a digital scale (Ohaus Corporation, Model Scout Pro SP401, Parsippany, NJ, USA, accuracy 0.1 g). The average weight of these five fruits was multiplied by the total number of commercial fruits produced on the 20 panicles to estimate the commercial fruit yield per 20 panicles [17].

### 4.8. Statistical Analysis

To compare the composition of floral visitors between both seasons, a non-metric multidimensional scaling analysis (NMDS) was carried out. Previously, a dissimilarity matrix was constructed based on the Bray–Curtis index, which considers the difference in the abundance of species between the samples. A permutational analysis of variance (PERMANOVA) with 999 permutations was applied to statistically test if the composition of floral visitors differed between both seasons. The NMDS was performed using the metaMDS function of the ‘vegan’ package [84]. The quality of the representation was evaluated by means of the stress value. The PERMANOVA uses the adonis function of the same package. Indicator species analyses were conducted using the ‘indicspecies’ package [85], to identify taxa significantly associated with each flowering season.

Generalized linear models (GLMs) with negative binomial distribution (with a logarithmic link function due to overdispersion) were used to evaluate the effect of flowering season, floral visitor group, and their interaction on the number of pollen grains deposited on the stigma. Generalized linear mixed models (GLMMs) were used to evaluate the effect of the flowering season on the richness and abundance of floral visitors, pollen limitation, production of commercial fruits, incidence of nubbins, quality of fruits, and yield. The abundance of the eight groups of floral visitors was analyzed separately and together. In all models, the flowering season was included as a predictor. Specifically, to compare the richness of floral visitors and the production of commercial fruits, a Poisson distribution and a logarithmic link function were used due to the nature of counting data, and the orchard pair was included as a random effect. To compare the total abundance of the different groups of floral visitors, a negative binomial distribution and a logarithmic link function (due to overdispersion) were used, and the time of day and orchard (nested within orchard pair) were included as random effects. The binomial distribution and a logit link function were used to compare the incidence of nubbins and pollen limitation (due to proportional data), with the orchard included as a random effect nested within the orchard pair. GLMMs with a negative binomial distribution (with a logarithmic link function due to overdispersion) were used to evaluate the effect of flowering season and pollination treatments (fixed factors) on the number of total (nubbins and commercial) and per-panicle commercial fruits. The panicle was included as a random effect nested within the tree, which was nested within the orchard and orchard pair. To compare the equatorial and polar diameters, the fresh weight of commercial fruits, and the yield of each tree, a gamma distribution and an identity linking function were used given the continuity of data, with the tree included as a random effect nested within the orchard and orchard pair. For each flowering season, a GLMM was performed to examine the relationship between yield and floral visitor abundance, including honeybees and all wild floral visitors (i.e., all non-honeybee floral visitors) and their interaction as predictors, while the yield was used as a response variable and orchards as a random effect. The two explanatory variables were standardized (mean = 0, SD = 1) using the ‘scale’ package, to avoid GLMM convergence issues. The spatial autocorrelation was estimated via Moran’s I test, using residuals from the models, implemented with the testSpatialAutocorrelation function from the ‘DHARMa’ package [86]. No significant spatial autocorrelation was detected (*p* > 0.05 in all cases, Table S5).

GLMs and GLMMs were adjusted with the glmmTMB function from the ‘glmmTMB’ package [87], and the significance was estimated using likelihood ratio tests (ANOVA) between the models that contained the predictor of interest and their respective null model (a simpler model without the predictor of interest) using a chi-square test. Random effects that did not improve model fit were excluded to obtain more parsimonious models. Each model was graphically validated using the ‘DHARMa’ package [86]. For a posteriori comparison, the ‘emmeans’ package with the Tukey method [88] and the ggemmeans function of the ‘ggeffects’ package were used to obtain the predicted means and their standard error of the models. All statistical analyses were performed with software R version 4.3.1 [89] and were conducted separately for each year to evaluate pollination patterns within each annual context and to avoid confounding seasonal flowering comparison with potential year effects.

## 5. Conclusions and Recommendations for Mango Producers

The findings of this study highlight that maintaining diverse pollinator communities can help support the pollination service in mango orchards in southern Mexico over time [90]. This not only allows mango producers to ensure fruit production through different seasons of flowering but also offers other important benefits, such as the ability to mitigate the effects of environmental variability and strengthen the resilience of the production system [90].

In this context, practices such as conserving nearby forest patches [28], maintaining semi-natural habitats within orchards [91], and reducing the use of herbicides, pesticides, and other agrochemicals could contribute to sustaining pollinator populations [92,93]. In addition, given that many of the wild pollinators of the ‘Ataulfo’ mango are dipterans, the incorporation of decomposing organic matter, such as litter, dung, or carrion, can serve to attract diverse species of pollinating flies into the orchards [94]. These strategies are not only expected to increase the productivity of the ‘Ataulfo’ mango crop under different seasons of flowering, but they will also contribute to the conservation of native forest and pollinators.

## Figures and Tables

**Table 1:** Pollination effectiveness (PE) of eight floral visitors in mango ‘Ataulfo’ during the off-season and natural flowering of 2023, based on the product of their abundances and the number of pollen grains deposited on the stigma per single visit.

Floral Visitor Group	Off-Season	Natural
Honeybees	1.63	26.33
Blowflies	6.69	1.96
Stingless bees	0.42	3.55
Yellow-banded wasps	2.21	0.48
Hoverflies	0.37	0.46
Other flies	0.24	0.45
Honey wasps	0.30	0.00
Other wasps	0.00	0.12
Total	11.86	33.34

## Data Availability

The original contributions presented in this study are included in the article. The data that support the findings of this study are available from figshare at https://doi.org/10.6084/m9.figshare.31282927 (accessed on 6 February 2026).

## Supplementary Materials

The following supporting information can be downloaded at https://www.mdpi.com/article/10.3390/plants15071124/s1, Figure S1: Richness of insect species that visited the ‘Ataulfo’ mango flowers during the off-season and natural flowering in the years 2022 and 2023 in the Costa Grande region, Guerrero, Mexico. The data represents the average ± standard error. The asterisks above the bars indicate significant differences between flowering seasons, according to *χ*^2^ tests followed by post hoc comparisons (* *p* < 0.05); Figure S2: Accumulated monthly precipitation (a) and average monthly temperature (b) during the off-season and natural flowering of the ‘Ataulfo’ mango in 2022 and 2023 in the Costa Grande region, Guerrero, Mexico. The climatic data were obtained from meteorological stations near the study area; Table S1: Abundance (AB^1^) of floral visitors of ‘Ataulfo’ mango flowers in nine orchards during off-season and natural flowering of 2022 and 2023 in Costa Grande, Guerrero, Mexico^2^; Table S2: Number of pollen grains deposited after a single visit (PD) by different insects on ‘Ataulfo’ mango flowers in natural and off-season flowering in Costa Grande, Guerrero, Mexico^1^; Table S3: Pollen limitation^1^ (mean ± sd) in ‘Ataulfo’ mango orchards during off-season and natural flowering in 2023 in the Costa Grande region, Guerrero, Mexico; Table S4: Pairwise geographic distances (in kilometers) between the nine studied mango orchards in Guerrero, Mexico, based on their centroid coordinates; Table S5: Results of Moran’s I tests for spatial autocorrelation of model residuals, evaluating the effect of floral visitor abundance on mango yield across off-season and natural flowering in ‘Ataulfo’ mango orchards during 2022 and 2023.
