# Supplementary material for: Wild Insects Contribute More to Mango Pollination and Yield than Exotic Honeybees During Induced Off-Season Flowering in Southern Mexico
Source: Plants (Basel). 2026 Apr 7;15(7):1124. doi: 10.3390/plants15071124 (PMC13074445; doi:10.3390/plants15071124)
Supplement: Supplementary file 1 [file plants-15-01124-s001.zip › plants-4155700-supplementary.pdf]

Supplementary material for:

Wild Insects Contribute More to Mango Pollination and Yield than Exotic Honeybees During Induced Off-Season Flowering in Southern Mexico

Rodrigo Lucas-García[1,2,*], *Víctor Rosas-Guerrero**[,2], *Eduardo Cuevas*[3] *and Carina Gutiérrez-Flores*[4]

Table S1. Abundance (AB[1]) of floral visitors of 'Ataulfo' mango flowers in nine orchards during off-season and natural flowering of 2022 and 2023 in Costa Grande, Guerrero, Mexico[2].

ORDER Family	Species	Group	2022			2023		
			Off-season	Natural	Total	Off-season	Natural	Total
HYMENOPTERA								
Apidae	*Apis mellifera*	Honeybees	574	2058	2632	758	1555	2313
	Stingless bee sp. 1	Stingless bees	15	199	214	43	107	150
	Frieseomelitta nigra	Honey wasps	34	64	98	9	19	28
	Stingless bee sp. 2	Stingless bees	0	0	0	0	5	5
Vespidae	*Polybia occidentalis*	Yellow-banded wasps	33	56	89	243	162	405
	Brachygastra azteca	Honey wasps	18	15	33	99	61	160
	Polistes instabilis	Other wasps	5	12	17	70	11	81
	Polistes dorsalis	Other wasps	0	0	0	9	5	14
	Vespid sp. 1	Other wasps	0	0	0	0	10	10
	Polistes major	Other wasps	2	1	3	4	1	5
	Vespid sp. 2	Other wasps	0	0	0	0	3	3
DIPTERA								
Calliphoridae	*Chrysomya megacephala*	Blowflies	121	15	136	291	295	586
	Compsomyiops sp.1	Blowflies	39	6	45	122	107	229
	Chrysomya sp. 2	Blowflies	26	1	27	65	53	118
	Cochliomyia sp. 1	Blowflies	12	2	14	43	21	64
	Chrysomya sp. 3	Blowflies	11	2	13	11	26	37
	Lucila sp. 1	Blowflies	7	2	9	6	7	13
Syrphidae	*Palpada vinetorum*	Hoverflies	56	51	107	30	97	127
	Palpada rufiventris	Hoverflies	8	17	25	12	36	48

	Palpada pusilla	Hoverflies	23	14	37	3	26	29
	Allograpta obliqua	Hoverflies	6	4	10	9	31	40
	Ornidia obesa	Hoverflies	9	2	11	6	25	31
	Palpada triangularis	Hoverflies	0	0	0	8	26	34
	Palpada mexicana	Hoverflies	5	10	15	1	17	18
	Ocyptamus aff. *lividus*	Hoverflies	7	2	9	8	9	17
	Pseudodorus clavatus	Hoverflies	1	2	3	5	15	20
	Taxomerus politus	Hoverflies	0	0	0	5	7	12
	Allograpta exótica	Hoverflies	1	2	3	1	7	8
	Copestylum aff. *pallen*	Hoverflies	0	0	0	0	11	11
	Palpada furcata	Hoverflies	1	4	5	0	4	4
	Taxomerus pictus	Hoverflies	0	0	0	0	5	5
	Ocyptamus dimidiatus	Hoverflies	0	0	0	0	4	4
	Copestylum macrocephalum	Hoverflies	0	0	0	0	2	2
	Taxomerus aff. *marginatus*	Hoverflies	0	0	0	0	2	2
	Ceriana macquarti	Hoverflies	0	0	0	0	1	1
Muscidae	*Musca domestica*	Other flies	37	6	43	68	33	101
	Muscid sp. 1	Other flies	6	4	10	1	57	58
	Muscid sp. 2	Other flies	0	0	0	57	6	63
Sarchophagidae	Sarcophagid sp. 1	Other flies	2	7	9	48	28	76
	Sarcophagid sp. 2	Other flies	1	1	2	4	9	13
Tabanidae	*Tabanus* sp. 1	Other flies	3	0	3	1	7	8
	Tabanus sp. 2	Other flies	0	0	0	1	4	5
	Tabanus sp. 3	Other flies	0	0	0	0	2	2
Bombyliidae	Bombyliid sp. 1	Other flies	0	0	0	0	3	3

Tachinidae	Tachinid sp. 1	Other flies	0	0	0	1	7	8
	Tachinid sp. 5	Other flies	0	0	0	0	7	7
	Tachinid sp. 2	Other flies	0	0	0	1	5	6
	Tachinid sp. 4	Other flies	0	0	0	0	2	2
	Tachinid sp. 3	Other flies	0	0	0	1	0	1
			1063	2559	3622	2044	2943	4987

[1]AB was estimated on one day at each orchard along three transects (60 × 2 m) for 10 min three times a day. [2]Floral visitors are sorted in descending order within each family according to their total AB from both years.

Table S2. Number of pollen grains deposited after a single visit (PD) by different insects on 'Ataulfo' mango flowers in natural and off-season flowering in Costa Grande, Guerrero, Mexico[1].

Order	Family	Species	Group	Natural		Off-season		Average PD
				n	PD	n	PD	
Hymenoptera	Apidae	*Frieseomelitta nigra*	Stingless bees	7	1.43	15	0.27	0.85
		Apis mellifera	Honeybees	29	0.52	29	0.14	0.33
		Stingless bee sp. 1	Stingless bees	-	-	22	0.32	0.32
		Plebeia sp. 1	Stingless bees	1	0.0	5	0.0	0.0
	Vespidae	*Polybia occidentalis*	Yellow-banded wasps	10	0.10	13	0.31	0.20
		Polistes instabilis	Other wasps	3	0.33	1	0.0	0.17
		Brachygastra azteca	Honey wasps	1	0.0	8	0.13	0.06
		Polistes dorsalis	Other wasps	2	0.0	-	-	0.0
		Polistes major	Other wasps	1	0.0	-	-	0.0
Diptera	Calliphoridae	*Chrysomya* sp. 2	Blowflies	4	0.25	9	1.22	0.74
		Chrysomya sp. 1	Blowflies	1	0.0	11	1.00	0.50
		Chrysomya megacephala	Blowflies	13	0.15	21	0.76	0.46
		Compsomyiops sp.1	Blowflies	5	0.40	4	0.25	0.33
		Cochliomyia sp. 1	Blowflies	8	0.25	7	0.29	0.27
		Lucila sp. 1	Blowflies	3	0.33	2	0.0	0.17
	Syrphidae	*Palpada mexicana*	Hoverflies	1	1.00	-	-	1.00
		Allograpta obliqua	Hoverflies	3	0.0	2	0.50	0.25
		Palpada vinetorum	Hoverflies	7	0.0	2	0.0	0.0
		Palpada pusilla	Hoverflies	3	0.0	-	-	0.0
		Copestylum sp. 1	Hoverflies	-	-	1	0.0	0.0
		Ornidia obesa	Hoverflies	2	0.0	-	-	0.0
		Pseudodorus clavatus	Hoverflies	2	0.0	-	-	0.0
		Ocyptamus sp. 1	Hoverflies	-	-	1	0.0	0.0
	Tachinidae	Tachinid sp. 6	Other flies	3	0.33	-	-	0.33
		Tachinid sp. 1	Other flies	4	0.25	-	-	0.25
		Tachinid sp. 2	Other flies	1	0.0	-	-	0.0
		Tachinid sp. 3	Other flies	1	0.0	-	-	0.0
	Tabanidae	*Tabanus* sp. 1	Other flies	2	0.50	-	-	0.50
		Diachlorus sp. 1	Other flies	1	0.0	-	-	0.0
	Sarchophagidae	Sarcophagid sp. 1	Other flies	3	0.0	3	0.33	0.17
		Sarcophagid sp. 2	Other flies	6	0.0	-	-	0.0
	Bombyliidae	Bombyliid sp. 1	Other flies	4	0.0	-	-	0.0
	Muscidae	*Musca domestica*	Other flies	-	-	9	0.0	0.0

	Muscid sp. 1	Other flies	2	0.0	2	0.0	0.0
	Muscid sp. 2	Other flies	-	-	4	0.0	0.0
**Total**			**133**		**171**		

[1]Floral visitors are sorted in descending order within each family according to their average PD from both flowering seasons.

Table S3. Pollen limitation (mean ± SD) in ‘Ataulfo’ mango orchards during off-season and natural flowering in 2023 in the Costa Grande region, Guerrero, Mexico.

**Orchard**	**Total fruits**		**Commercial fruits**	
	**Off-season**	**Natural**	**Off-season**	**Natural**
bomba	0.39 ± 0.26	0.14 ± 0.12	0.93 ± 0.14	0.98 ± 0.05
cornelio	0.66 ± 0.36	0.38 ± 0.41	0.79 ± 0.19	0.93 ± 0.06
cuatas	0.79 ± 0.10	0.89 ± 0.09	0.80 ± 0.13	0.88 ± 0.10
fogata	0.78 ± 0.14	0.51 ± 0.25	0.94 ± 0.05	0.64 ± 0.25
limon	0.86 ± 0.14	0.36 ± 0.38	0.93 ± 0.05	0.94 ± 0.07
ojo_agua	0.00 ± 0.00	0.59 ± 0.03	0.74 ± 0.15	0.51 ± 0.07
playa	0.25 ± 0.29	0.25 ± 0.50	0.95 ± 0.07	0.98 ± 0.05
san_luis	0.80 ± 0.13	0.87 ± 0.08	0.85 ± 0.14	0.87 ± 0.08
tarros	0.20 ± 0.23	0.10 ± 0.21	0.95 ± 0.05	1.00 ± 0.00

Table S4. Pairwise geographic distances (in kilometers) between the nine studied mango orchards in Guerrero, Mexico, based on their centroid coordinates.

**Orchard**	2	3	4	5	6	7	8	9
1. cuatas	2.2	4.7	4.8	9.9	10.0	16.7	27.2	40.7
2. cornelio		6.6	3.2	12.0	7.8	14.6	25.0	38.5
3. san_luis			9.5	7.5	13.9	20.3	31.2	44.3
4. tarros				13.7	6.9	13.7	23.3	37.0
5. playa					19.9	26.6	36.9	50.5
6. ojo_agua						6.8	17.3	30.6
7. limon							11.3	24.0
8. fogata								14.0

Table S5. Results of Moran's I tests for spatial autocorrelation of model residuals, evaluating the effect of floral visitor abundance on mango yield across off-season and natural flowering in 'Ataulfo' mango orchards during 2022 and 2023.

**Response variable**	**2022**		**2023**	
	**Moran I**	***P***	**Moran I**	***P***
Off-season	-0.206	0.952	-0.141	0.860
Natural	-0.350	0.145	-0.173	0.605

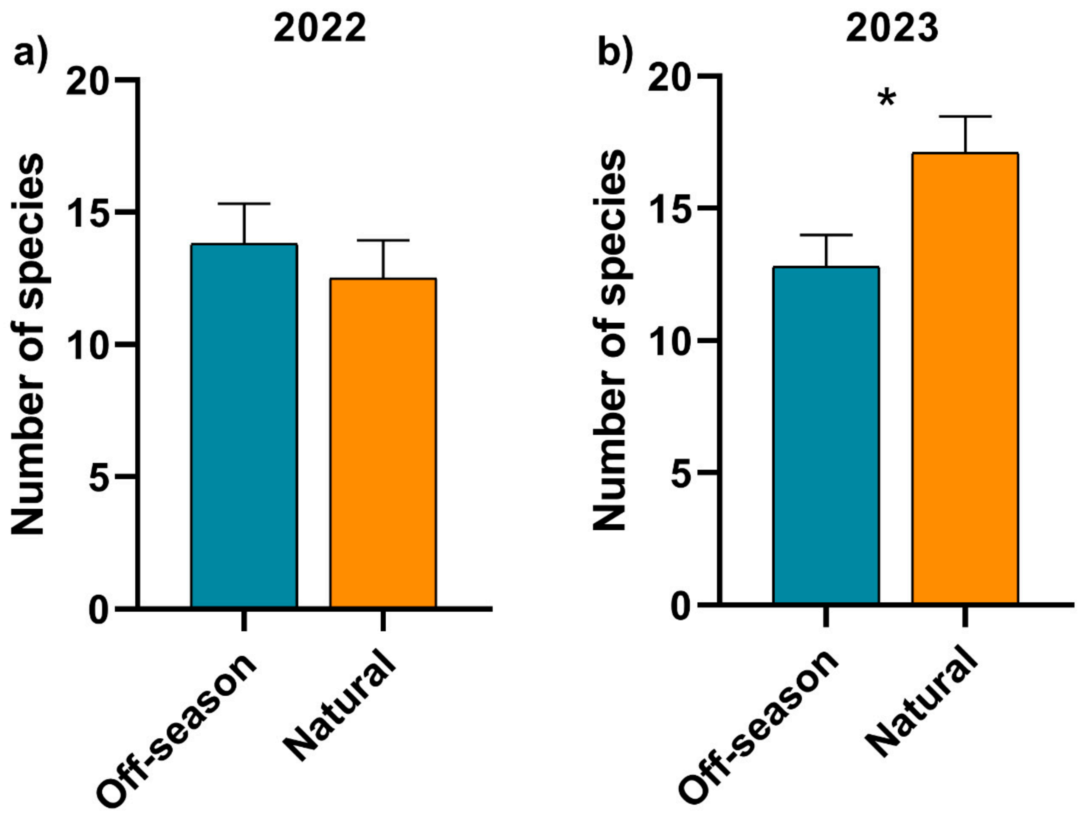

Figure S1. Richness of insect species that visited the 'Ataulfo' mango flowers during the off-season and natural flowering in the years 2022 and 2023 in the Costa Grande region, Guerrero, Mexico. The data represents the average ± standard error. The asterisks above the bars indicate significant differences between flowering seasons, according to χ^2 tests followed by post hoc comparisons (* $P < 0.05$).

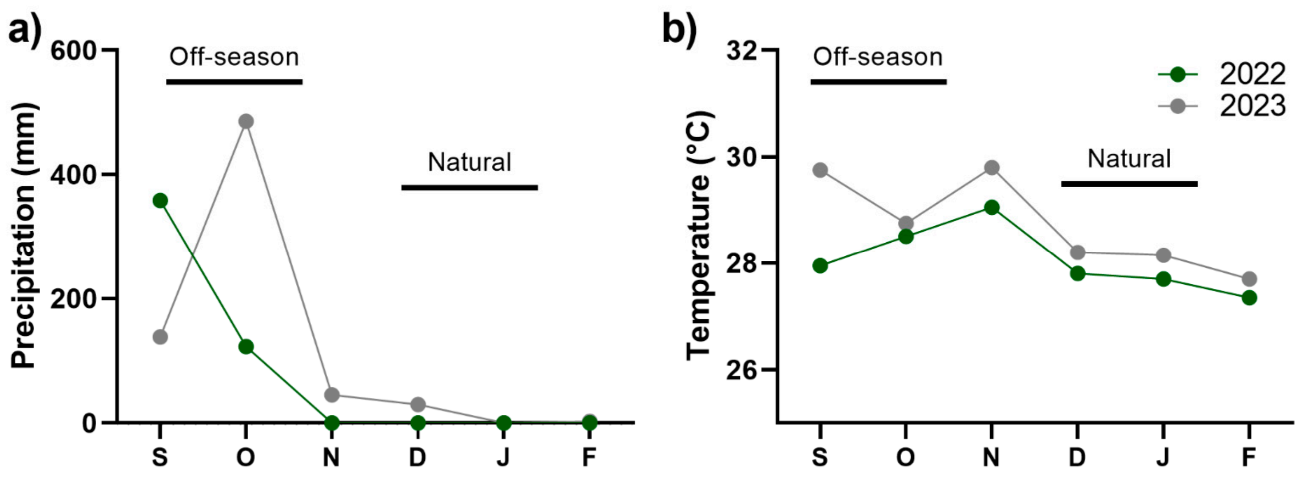

Figure S2. Accumulated monthly precipitation (a) and average monthly temperature (b) during the off-season and natural flowering of the 'Ataulfo' mango in 2022 and 2023 in the Costa Grande region, Guerrero, México. The climatic data were obtained from meteorological stations near the study area.
